# Supplementary material for: Ultrafiltration Membranes Functionalized with Copper Oxide and Zwitterions for Fouling Resistance
Source: Membranes (Basel). 2022 May 23;12(5):544. doi: 10.3390/membranes12050544 (PMC9145826; doi:10.3390/membranes12050544)
Supplement: Supplementary file 1 [file membranes-12-00544-s001.zip › membranes-1720891-supplementary.pdf]

membranes

Supplementary Material

Ultrafiltration Membranes Functionalized with Copper Oxide and Zwitterions for Fouling Resistance

Cannon Hackett [1], Mojtaba Abolhassani [1], Lauren F. Greenlee [2] and Audie K. Thompson [1,*]

[1] Ralph E. Martin Department of Chemical Engineering, University of Arkansas, Fayetteville, AR 72701, USA; cjhacket@uark.edu (C.H.); Abolhassani.mo@gmail.com (M.A.)
[2] Department of Chemical Engineering, Pennsylvania State University, University Park, PA 16802, USA; lfg5271@psu.edu
* Correspondence: akt022@uark.edu; Tel.: +1-479-575-3857

Citation: Hackett, C.; Abolhassani, M.; Greenlee, L.F.; Thompson, A.K. Ultrafiltration Membranes Functionalized with Copper Oxide and Zwitterions for Fouling Resistance. *Membranes* **2022**, *12*, 544. https://doi.org/10.3390/membranes12050544

Academic Editor: Teresa Poerio

Received: 25 April 2022
Accepted: 20 May 2022
Published: 23 May 2022

Publisher's Note: MDPI stays neutral with regard to jurisdictional claims in published maps and institutional affiliations.

Copyright: © 2022 by the authors. Licensee MDPI, Basel, Switzerland. This article is an open access article distributed under the terms and conditions of the Creative Commons Attribution (CC BY) license (https://creativecommons.org/licenses/by/4.0/).

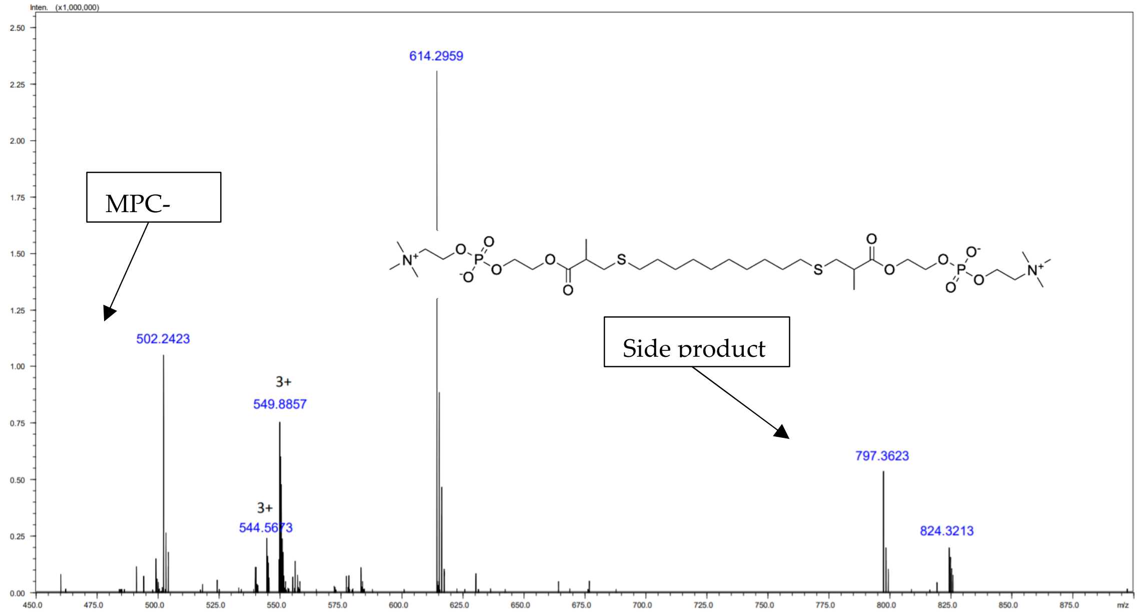

Figure S1. Mass spectrometry results for the MPC-SH synthesis, with a reaction time of 8 hours. The side product structure is shown on the right.

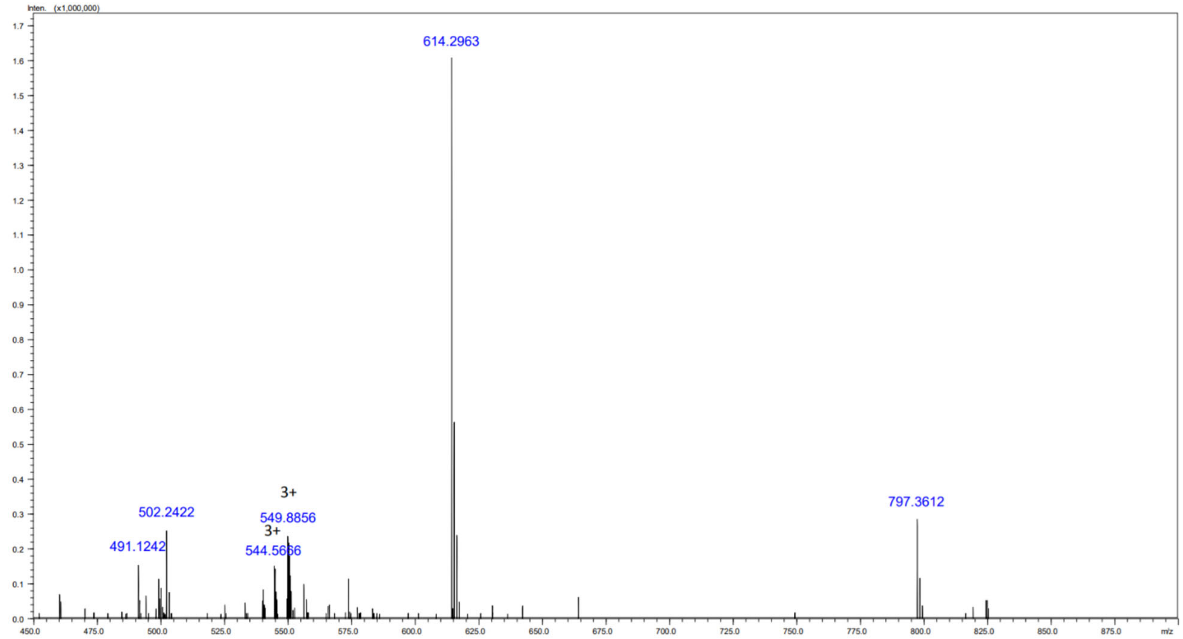

Figure S2. Mass spectrometry results for the MPC-SH synthesis, with a reaction time of 20 hours.

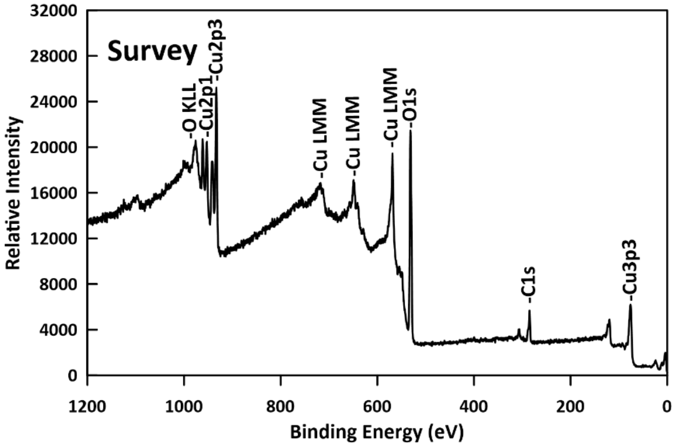

Figure S3. XPS survey spectrum for CuO NPs.

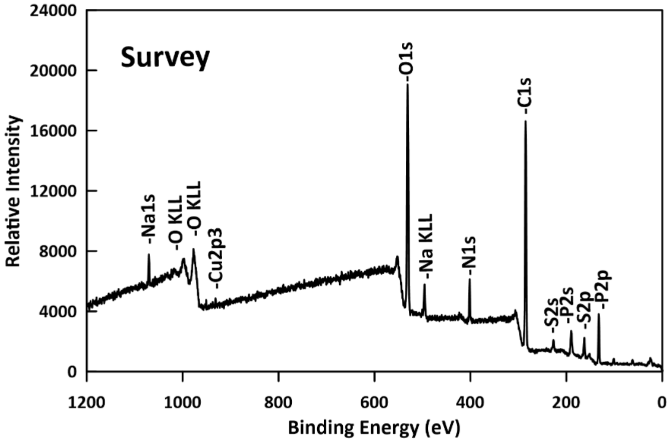

Figure S4. XPS survey spectrum for CuO/MPC NPs.

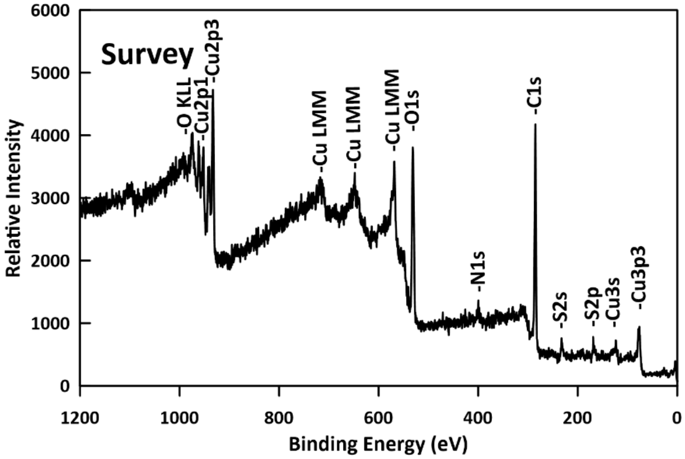

Figure S5. XPS survey spectrum for PES/PDA/MPC/CuO membrane.
